# A Novel Pathway of Functional microRNA Uptake and Mitochondria Delivery

**DOI:** 10.1002/advs.202300452

**Published:** 2023-06-25

**Authors:** Jiachen Liu, Weili Li, Jianfeng Li, Eli Song, Hongwei Liang, Weiwei Rong, Xinli Jiang, Nuo Xu, Wei Wang, Shuang Qu, Shouyong Gu, Yujing Zhang, Chen‐ Yu Zhang, Ke Zen

**Affiliations:** ^1^ State Key Laboratory of Pharmaceutical Biotechnology Nanjing University School of Life Sciences Nanjing Jiangsu 210093 China; ^2^ The Laboratory of Biological Electron Microscopy and Structural Biology Centre for Biological Imaging Institute of Biophysics Chinese Academy of Sciences 15 Datun Road, Chaoyang District Beijing 100101 China; ^3^ School of Life Science and Technology China Pharmaceutical University 639 Longmian Avenue Nanjing Jiangsu 211198 China; ^4^ Institute of Geriatric Medicine Jiangsu Province Geriatric Hospital Nanjing Jiangsu China

**Keywords:** miRNA uptake, mitochondria, PNPT1, RNA phase separation

## Abstract

Extracellular microRNAs (miRNAs) play a critical role in horizontal gene regulation. Uptake of extracellular miRNAs by recipient cells and their intracellular transport, however, remains elusive. Here RNA phase separation is shown as a novel pathway of miRNA uptake. In the presence of serum, synthetic miRNAs rapidly self‐assembly into ≈110 nm discrete nanoparticles, which enable miRNAs’ entry into different cells. Depleting serum cationic proteins prevents the formation of such nanoparticles and thus blocks miRNA uptake. Different from lipofectamine‐mediated miRNA transfection in which majority of miRNAs are accumulated in lysosomes of transfected cells, nanoparticles‐mediated miRNA uptake predominantly delivers miRNAs into mitochondria in a polyribonucleotide nucleotidyltransferase 1(PNPT1)‐dependent manner. Functional assays further show that the internalized miR‐21 via miRNA phase separation enhances mitochondrial translation of cytochrome b (CYB), leading to increase in adenosine triphosphate (ATP) and reactive oxygen species (ROS) reduction in HEK293T cells. The findings thus reveal a previously unrecognized mechanism for uptake and delivery functional extracellular miRNAs into mitochondria.

## Graphical abstract

In the presence of serum, microRNAs (miRNAs) spontaneously form ≈110 nm nanoparticles, mediated bycationic proteins. When depleted, miRNA uptake is blocked. Unlike lysosome‐located, nanoparticle‐delivere mechanism are preferentially delivered to mitochondria of recipient cells by PNPT1.

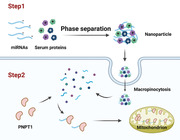

## 1 Introduction

Given their ability to interact with a broad, yet specific set of target genes through a base‐pairing mechanism, miRNAs significantly contribute to the gene regulation network.^[^ ^1^ ^]^ It is widely accepted that almost all cell types can produce and secrete miRNAs. As the extracellular miRNAs released by host cells can enter the recipient cells where they serve as a novel class of signaling molecules to modulate the expression of target genes and the function of recipient cells,^[^ ^2^, ^3^ ^]^ miRNAs play a critical role in horizontal gene regulation.^[^ ^4^ ^]^ Given the role of extracellular miRNAs in the recipient cells, extensive studies have been carried out to find out how extracellular miRNAs enter the recipient cells. It has been shown that multiple pathways, including endocytosis, phagocytosis, micropinocytosis, and macropinocytosis, are involved in uptake of miRNAs by various recipient cells.^[^ ^5^, ^6^ ^]^ However, the mechanisms of transferring functional miRNAs from host to recipient cells remain incompletely understood.

As ≈22 nt hydrophilic oligonucleotides, miRNA is not able to directly cross cell surface membranes. Previous studies demonstrate that there are three major pathways for delivering extracellular miRNAs into the recipient cells. First, miRNA uptake by recipient cells is mediated by extracellular vesicles (EVs), particularly exosomes.^[^ ^7^, ^8^ ^]^ In response to various stimuli, cells can selectively pack miRNAs into exosomes and release them via exocytosis pathway.^[^ ^2^ ^]^ Uptake of EV‐encapsulated miRNAs by recipient cells is dependent upon the type of recipient cells.^[^ ^7^ ^]^ For different recipient cells, EV‐miRNA uptake can be carried out via endocytosis, phagocytosis, and micropinocytosis.^[^ ^9^, ^10^, ^11^ ^]^ Utilizing selective inhibitors and specific endocytosis markers, Tian et al. demonstrated exosomal miR‐21 uptake by bone marrow‐derived mesenchymal stromal cell through clathrin‐mediated endocytosis and macropinocytosis pathway.^[^ ^12^ ^]^ Second, in addition to the encapsulation by EVs, a significant portion of extracellular miRNAs are in various free‐floating complexes associated with RNA‐binding proteins, such as Argonaute 2 (AGO2) ^[^ ^13^, ^14^, ^15^ ^]^ or nucleophosmin.^[^ ^16^ ^]^ Uptake of the extracellular miRNAs by recipient cells has been shown to be mediated by these RNA‐binding protein.^[^ ^13^, ^14^, ^15^, ^16^, ^17^ ^]^ For instance, AGO2‐bound miRNAs were reported to be taken up by recipient cells via neurophilin‐1 (17). Third, extracellular miRNAs can also be associated with high‐density lipoproteins (HDL) and low‐density lipoproteins (LDL), two highly abundant miRNA carriers.^[^ ^18^, ^19^, ^20^ ^]^ As one of the most critical miRNA carriers, these lipoproteins can mediate extracellular miRNA uptake in various cells.^[^ ^18^, ^21^ ^]^ For example, Vickers et al. found that the profile of HDL‐associated miRNAs was different from that in exosomes, and that HDL‐mediated miR‐223 uptake could repress cholesterol uptake by directly downregulating the scavenger receptor BI.^[^ ^21^ ^]^ Frank et al. showed that uptake of tumor cell‐derived miR‐375 was independent of exosomes but mediated by LDL.^[^ ^18^ ^]^ Moreover, they identified CD36 as a receptor for mediating the uptake of LDL‐miR‐375 complex by tumor‐associated macrophage.^[^ ^18^ ^]^ These extracellular miRNA pathways described above, however, still cannot explain the uptake of extracellular miRNAs that are neither encapsulated in EVs nor associated with RNA‐binding proteins or lipoproteins. In exploring the function of exogenous miRNAs in mammalian cells, we found rapid uptake of food‐derived miRNAs by human or murine intestinal epithelial cells, and that these exogenous miRNAs were not encapsulated in EVs or associated with RNA‐binding proteins or lipoproteins.^[^ ^22^ ^]^

In the present study, we demonstrate a novel pathway of general rapid miRNA uptake in various cell types. Different from uptake mediated by EVs or various miRNA carriers, this miRNA uptake pathway is dependent upon small RNA phase separation mediated by serum cationic proteins. More importantly, instead of sorting to lysosomes for degradation, the internalized miRNAs through this unique pathway are mainly delivered into mitochondria of recipient cells where they may interact with their target genes and thus modulate mitochondrial functions.

## 2 Results

### 2.1 miRNAs Are Taken by Various Cells in the Presence of Serum and Predominantly Delivered to Mitochondria

**Figure 1:**
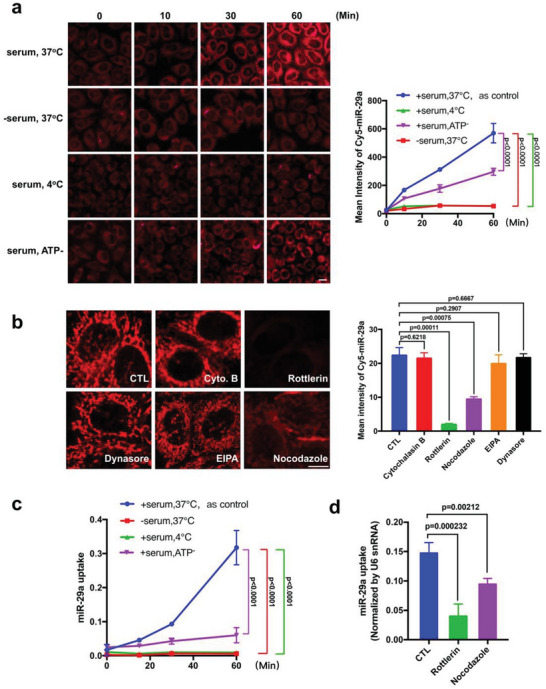
Direct uptake of extracellular miRNAs by recipient cells in the presence of serum. a) Left: time course of Cy5‐miR‐29a uptake by HeLa cell under various conditions. Right: quantification of Cy5‐miR‐29a uptake by HeLa cells with various treatment. b) Left: inhibition of Cy5‐miR‐29a uptake by various cytoskeleton disrupting reagents. Right: quantification of inhibitory effect of reagents on Cy5‐miR‐29a uptake. c) Time‐dependent miR‐29a uptake by HeLa cells quantified by RT‐qPCR under various conditions. d) Inhibition of miR‐29a uptake by rottlerin and nocodazole validated by RT‐qPCR. In fluorescence image quantification analysis, 10–12 image fields from 3 independent experiments were analyzed. In RT‐qPCR assay, experiments were repeated four times and samples at each time point were triplicated. In panel a, scale bar: 20 µm. In panel b, scale bar: 10 µm. Statistical differences between groups in panel a and c were assessed by two‐way ANOVA. Statistical differences between groups in panel b and d were assessed by independent‐samples *t*‐test. Data with *P*‐value < 0.05 were considered statistically significant.

To explore the interaction between extracellular miRNAs and host cells, we incubated HeLa cells with fluorescent 5′‐Cy5‐miR‐29a (miR‐29a attaching fluorophore Cyanine 5 (Cy5) at the 5′‐terminus) at 37 °C in the presence of 10% fetal bovine serum (FBS). To our surprise, we observed that, instead of just adherent to the cell surface, 5′‐Cy5‐miR‐29a rapidly entered the cells in a time‐dependent manner (**Figure** **1**a, left). Quantitative analysis of cell‐associated fluorescence confirmed the time‐dependent fluorescent miR‐29a uptake by HeLa cells (Figure 1a, right). Uptake of miR‐29a by HeLa or other cell lines were validated using 5′‐Cy3‐miR‐29a (miR‐29a attaching fluorophore Cyanine 3 (Cy3) at the 5′‐terminus) (Extended data, Figure S1a, Supporting Information). As a control, incubating cells with fluorophore Cy3 or Cy5 alone displayed no cell‐associated fluorescence (Extended data, Figure S1b,c, Supporting Information), suggesting that miR‐29a uptake by various cells is independent of fluorophore modification. As expected, entry of miR‐29a‐Cy5 into HeLa cells completely blocked at low temperature or after ATP depletion (Figure 1a), confirming that miRNA uptake by the recipient cells is an active process. Interestingly, uptake of 5′‐Cy5‐miR‐29a by HeLa cells at 37 °C was also abolished in the absence of serum (Figure 1a), suggesting that uptake of 5′‐Cy5‐miR‐29a in HeLa cells at 37 °C is mediated by serum. The uptake of miRNAs in the presence of serum was further validated in different cell types including HEK293T, A549, U87MG, Min6, and SGC‐7901, using fluorescent Cy5‐ or Cy3‐labeled miRNAs (Extended data, Figure S1d,e, Supporting Information). As disrupting cellular microfilament networks can block various endocytosis, micropinocytosis, and macropinocytosis,^[^ ^23^, ^24^ ^]^ we further analyze the uptake of 5′‐Cy5‐miR‐29a in the presence of serum after treating HeLa cells with cytochalasin B, an inhibitor of actin polymerization,^[^ ^25^ ^]^ rottlerin, an inhibitor of Rab34‐mediated macropinocytosis,^[^ ^26^, ^27^ ^]^ dynasore, an inhibitor of dynamin GTPase activity that blocks clathrin‐mediated endocytosis,^[^ ^28^ ^]^ ethylisopropylamiloride (EIPA), an autophagy enhancer through blocking Na^+^/H^+^‐exchange,^[^ ^29^ ^]^ nocodazole, a microtubule‐disrupting^[^ ^30^ ^]^ or other cytoskeleton disrupting reagents. After 1 h incubation, we found that cellular uptake of 5′‐Cy5‐miR‐29a was strongly prevented by rottlerin and nocodazole, whereas other reagents displayed no inhibition on 5′‐Cy5‐miR‐29a uptake (Figure 1b). This result suggests that uptake of 5′‐Cy5‐miR‐29a is likely via the pathway of microtubule‐dependent fluid phase macropinocytosis, which is in agreement with the notion that miRNA uptake in the presence of serum is an active process and dependent upon dynamic arrangement of the cellular microfilament network.

The uptake of extracellular miRNA by HeLa cells in the presence of serum was further validated using synthetic miR‐29a. As shown by qRT‐PCR analysis (Figure 1c,d), miR‐29a was rapidly taken up by HeLa cells at 37 °C in the presence of serum, whereas the time‐dependent miR‐29a uptake was abolished in the absence of serum, low temperature or ATP depletion (Figure 1c). Similarly, blockade of macropinocytosis process in HeLa cells by rottlerin and nocodazole markedly reduced miR‐29a uptake (Figure 1d).

**Figure 2:**
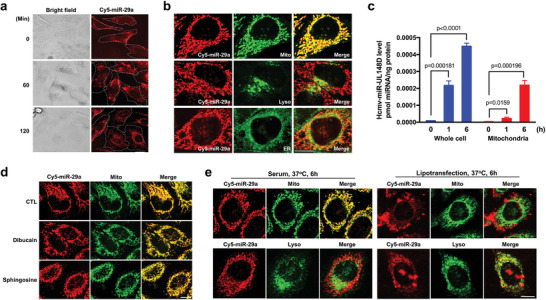
Selective delivery of internalized fluorescent miRNAs into mitochondria of recipient cells. a) Trace of internalized Cy5‐miR‐29a in HeLa cells for 2 h after initial uptake for 15 min. Cell boundary was indicated by white dot line. b) Double staining subcellular structures and 5′‐Cy5‐miR‐29a. c) RT‐qPCR analysis of exogenous hcmv‐miR‐UL148D in whole cell lysate and mitochondria at different time points (0, 1, 6 h) of incubation. d) Distribution of the internalized Cy5‐miR‐29a after disrupting mitochondrial membrane potential by Dibucaine and Sphingosine. e) Intracellular localization of Cy5‐miR‐29a after entering the cell through serum‐mediated pathway or direct transfection. HeLa cells were incubated with Cy5‐miR‐29a in the presence of 10% FBS or transfected with Cy5‐miR‐29a using Lipotransfectamine. After three washes, intracellular localization Cy5‐miR‐29a was observed at 6 h post‐treatment. Scale bars: 10 µm. The experiments were repeated three times and samples in each time point of RT‐qPCR assay were triplicated. Statistical differences in panel c were assessed by independent‐samples *t*‐test. Data with *P*‐value < 0.05 were considered statistically significant.

Next we examined the intracellular location of the internalized 5′‐Cy5‐miR‐29a. In this experiment, HeLa cells were incubated with 5′‐Cy5‐miR‐29a in the presence of 10% FBS 37 °C for 15 min, and then washed off the unbound 5′‐Cy5‐miR‐29a and continuously incubated for 120 min. As shown in **Figure** **2**a, 5′‐Cy5‐miR‐29a mainly located around the peripheral area inside cells after 15 min incubation, demonstrating a rapid internalization of 5′‐Cy5‐miR‐29a. Strikingly, tracing the internalized 5′‐Cy5‐miR‐29a for 60 and 120 min showed that the internalized 5′‐Cy5‐miR‐29a was predominantly transported to mitochondria‐like subcellular structures. Double staining subcellular structures with specific markers confirmed that internalized 5′‐Cy5‐miR‐29a after 6 h incubation was primarily delivered to mitochondria (mito) but not lysosomes (lyso) or endoplasmic reticulum (ER) (Figure 2b; Extended data, Figure S2a, Supporting Information). To validate the fluorescent miR‐29a tracing result, we incubated HeLa cells with hcmv‐miR‐UL148D, an exogenous miRNA encoded by human cytomegalovirus,^[^ ^31^ ^]^ in the presence of 10% FBS for 0, 1, and 6 h, and then examined the subcellular distribution of hcmv‐miR‐UL148D by RT‐qPCR with specific probe. As shown in Figure 2c, levels of hcmv‐miR‐UL148D in whole cell and mitochondria of HeLa cells were time‐dependently increased, however, the majority of internalized hcmv‐miR‐UL148D was detected in mitochondria after 6 h incubation.

Given that alteration of mitochondria membrane potential can change mitochondria shape and localization,^[^ ^32^ ^]^ we examined the intracellular localization of 5′‐Cy5‐miR‐29a following disruption of mitochondria membrane potential by Dibucaine and Sphingosine.^[^ ^33^ ^]^ As shown in Figure 2d, treatment with Dibucaine and Sphingosine did not alter the colocalization of internalized 5′‐Cy5‐miR‐29a with mitochondria tracker in HeLa cells after 6 h incubation. The cellular localization of internalized 5′‐Cy5‐miR‐29a in the presence of 10% FBS was also compared to that through lipofectamine transfection (Figure 2e). Different from lipofectamine transfection in the absence of serum, in which most 5′‐Cy5‐miR‐29a was observed in lysosomes at 6 h post‐transfection, miRNA uptake in the presence of serum mainly transported the internalized 5′‐Cy5‐miR‐29a into mitochondria after 6 h incubation at 37 °C (Figure 2e). To exclude the possibility that mitochondria delivery of internalized miRNA via serum‐mediated uptake may be restricted to certain miRNA or cell types, we tested various cell lines (A549, HEK293T, U87MIG, MIN6, and SGC‐7901) using different small RNAs, including miR‐21‐5p, miR‐122, miR‐1‐3p mimics, hcmv‐miR‐UL148D, and double‐strand miR‐29a. (The sequences were listed in Extended data, Table S1, Supporting Information.) We found that, without exception, all these internalized small RNAs were predominantly delivered into mitochondria in different cells (Extended data, Figure S2b,c, Supporting Information).

### 2.2 Uptake of miRNA Is Dependent upon RNA Phase Separation Mediated by Serum Cationic Proteins

**Figure 3:**
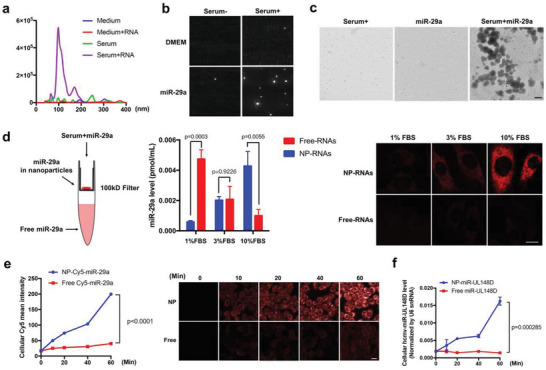
Uptake of extracellular miRNA in the presence of serum is dependent upon the formation of miRNA nanoparticles. a,b) Formation of ≈110 nm miR‐29a nanoparticles after mixing with 10% MV‐free FBS assessed by Nanosight. c) TEM images of 10% MV‐free serum, miR‐29a, and mixture of miR‐29a and 10% MV‐free serum. Scale bar: 200 nm. d) Left: schematic description of separating free miR‐29a (free miR‐29a) from miR‐29a in nanoparticles (NP‐miR‐29a) via centrifugation with a 100 kD cut‐off filter. Middle: NP‐miR‐29a or free‐miR‐29a level after mixing miR‐29a with 1%, 3% or 10% MV‐free FBS for 5 min. Right: uptake of free Cy5‐miR‐29a and NP‐Cy5‐miR‐29a formed in the presence of 1%, 3% or 10% MV‐free FBS by HeLa cells at 1 h post‐treatment. Scale bar: 10 µm. e) Dynamic uptake of free Cy5‐miR‐29a or NP‐Cy5‐miR‐29a formed in the presence of 10% MV‐free FBS by HeLa cells assessed by fluorescence imaging. Scale bar: 20 µm. f) Uptake of free miR‐29a or NP‐miR‐29a formed in the presence of 10% MV‐free FBS by HeLa cells assessed by RT‐qPCR. In fluorescence image quantification analysis, 10–12 image fields from 3 independent experiments were analyzed. In RT‐qPCR assay, the experiments were repeated three times and samples at each time point were triplicated. Statistical differences between samples in panel e and f were assessed by paired *t*‐test, while statistical differences in panel d were analyzed by independent‐samples *t*‐test. Data with *P*‐value < 0.05 were considered statistically significant.

As uptake of miRNA requires the presence of serum, our initial hypothesis was that certain serum proteins might serve as miRNA receptor(s) or carriers for mediating the miRNA uptake by the recipient cells. However, when we denatured the serum proteins by heat treatment (60 °C, 1 h) prior to the incubation of HeLa cells with exogenous hcmv‐miR‐UL148D, we found that hcmv‐miR‐UL148D uptake by HeLa cells was not affected by serum denature (Extended data, Figure S3, Supporting Information). As previous studies suggest that RNA phase separation, such as formation of nanoparticles, may facilitate the entry of RNA into cells,^[^ ^34^ ^]^ we examined whether miRNA phase separation occurred in the presence of serum. In this experiment, 50 pmol mL^−1^ synthetic miR‐29a was mixed with 10% FBS in DMEM and miRNA phase separation was monitored using Nanosight. Prior to this experiment, FBS was ultra‐centrifuged at 120 000 × *g* for 2 h to remove various MVs,^[^ ^8^, ^35^ ^]^ and then filtered (4000 × *g* for 20 min) using a 100 kD cutoff ultrafiltration tube, which depleted proteins larger than 100 kD. The Nanosight results clearly indicated that miR‐29a rapidly formed numerous ≈110 nm nanoparticles in the presence of 10% MV‐free FBS, whereas no nanoparticles were detected in the DMEM containing miR‐29a or MV‐free serum alone (**Figure** **3**a,b). The formation and size of miR‐29a nanoparticles in the presence of 10% MV‐free serum were further analyzed by transmission electron microscope (TEM), which confirmed the formation of ≈110 nm nanoparticles after mixing miR‐29a with 10% MV‐free FBS (Figure 3c). To test whether miRNA uptake by recipient cells relies on the formation of miRNA nanoparticles in the presence of MV‐free FBS, we separated the miR‐29a in nanoparticles (NP‐miR‐29a) from “free” miR‐29a using centrifugation with a 100 kD cutoff filter (Figure 3d, left). The mixture of miR‐29a with 1%, 3% or 10% MV‐free FBS in DMEM was uploaded separately into the column, which was subjected to centrifugation (4000 × *g*, 20 min). The “free” miR‐29a and majority of serum proteins were filtered through and collected at the bottom fraction while the NP‐miR‐29a was harvested in the top chamber. As shown in Figure 3d, middle, the RT‐qPCR assay showed that miRNA nanoparticles were rapidly formed after mixing miR‐29a with MV‐free FBS of various concentration. At 50 pmol mL^−1^ concentration, the percentage of NP‐miR‐29a steadily increased when the concentration of MV‐free FBS increased from 1% to 10%. In groups of miR‐29a mixing with of 1%, 3%, and 10% MV‐free FBS, the percentage of NP‐miR‐29a was 20%, 50%, and 80%, respectively.

We next investigated whether the nanoparticle formation in the presence of serum serves as the mechanism by which miRNAs were taken up by the recipient cells. In this experiment, both 5′‐Cy5‐miR‐29a and hcmv‐miR‐UL148D were used. Various miRNAs were mixed with 1%, 3% or 10% MV‐free FBS in DMEM and then separated into the nanoparticle fraction and the nanoparticle‐free fraction, respectively. As shown in Figure 3d, right, incubating HeLa cells with free miR‐29a or NP‐miR‐29a isolated from 1%, 3% or 10% MV‐free FBS group for 2 h confirmed that only NP‐miR‐29a but not free‐miR‐29a was taken up by HeLa cells. No uptake of free miR‐29a was detected even when high concentration of miR‐29a was added. HeLa cells were then incubated with 5′‐Cy5‐miR‐29a or hcmv‐miR‐UL148D in nanoparticle and nanoparticle‐free fractions at 37 °C for various time points. In this experiment, 10% MV‐free FBS was used to mix with miRNAs. The miRNA uptake by HeLa cells was assessed via fluorescence imaging system (5′‐Cy5‐miR‐29a) and qRT‐PCR assay (for hcmv‐miR‐UL148D), respectively. As shown in Figure 3e, cell‐associated fluorescence quantification indicated that only 5′‐Cy5‐miR‐29a in nanoparticle fraction entered the cells and uptake of NP‐5′‐Cy5‐miR‐29a by HeLa cells was in a time‐dependent manner. No uptake of free 5′‐Cy5‐miR‐29a was observed in HeLa cells. Similar results were obtained in the uptake of hcmv‐miR‐UL148D assayed by RT‐qPCR (Figure 3f). As shown, the NP‐hcmv‐miR‐UL148D but not free hcmv‐miR‐UL148D was time‐dependently taken up by HeLa cells.

**Figure 4:**
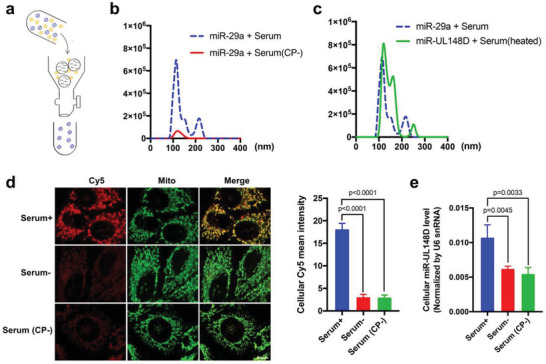
Formation of small RNA nanoparticles is mediated by serum cationic proteins. a) Schematic description of depletion of serum cationic proteins via ion‐exchange column. b) Depletion of serum cationic proteins completely blocked the formation of miR‐29a nanoparticles (red line). c) Denature of serum proteins via heating treatment (60 °C) did not affect the formation of small RNA nanoparticles (green line). d) Uptake of Cy5‐miR‐29a by HeLa cells in the presence of 10% MV‐free FBS (Serum+) or 10% cationic protein‐depleted, MV‐free FBS (serum CP‐) assessed by direct fluorescent imaging. Cy5‐miR‐29a uptake by HeLa cells in the absence of MV‐free FBS (Serum‐) served as negative control. e) Uptake of hcmv‐miR‐148D by HeLa cells under Serum‐, Serum+ or Serum CP‐ conditions assessed by RT‐qPCR. Scale bar: 10 µm. In fluorescence image quantification analysis, 10–12 image fields from 3 independent experiments were analyzed. In RT‐qPCR assay, the experiments were repeated four times and samples in each time point of RT‐qPCR assay were triplicated. Data in this experiment were analyzed by independent‐samples *t*‐test. Data with *P*‐value < 0.05 were considered statistically significant.

Previous studies have shown that cationic proteins or peptides such as polyarginine (R9) are involved in formation of large RNA nanoparticles.^[^ ^36^, ^37^ ^]^ Given that serum contains various cationic proteins derived from basophil and eosinophils,^[^ ^38^ ^]^ we next tested whether the cationic proteins in serum are responsible for miRNA phase separation, leading to formation of miRNA nanoparticles. As shown in **Figure** **4**a, positively charged proteins in MV‐free FBS were depleted using an ion exchange chromatography. As expected, MV‐free FBS with cationic protein depletion failed to mediate miRNA phase separation. When miR‐29a was mixed with 10% MV‐free FBS in which cationic proteins had been depleted, no nanoparticles of miR‐29a were detected (Figure 4b). In line with our previous observation that denatured serum was able to mediate miRNA uptake (Extended data, Figure S3, Supporting Information), mixing hcmv‐miR‐UL148D with 10% heat‐treated MV‐free FBS (60 °C, 1 h) also formed hcmv‐miR‐UL148D nanoparticles at a size of ≈110 nm (Figure 4c). Supporting the notion that formation of RNA nanoparticles in the presence of serum is the key for miRNA uptake by recipient cells, depleting cationic proteins in MV‐free FBS prevented the formation of miRNA nanoparticles, thus abolished the serum‐mediated uptake of 5′‐Cy5‐miR‐29a and hcmv‐miR‐UL148D by HeLa cells assessed by fluorescence imaging (Figure 4d) and RT‐qPCR (Figure 4e), respectively.

### 2.3 PNPT1 Mediates the Delivery of Internalized miRNA to Mitochondria of Recipient Cells

**Figure 5:**
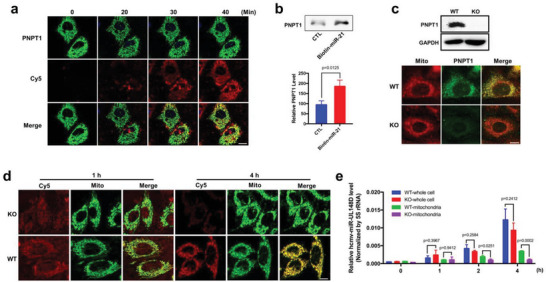
PNPT1 delivers the internalized miRNAs to mitochondria of recipient cells. a) Colocalization of internalized Cy5‐miR‐21 (red) with EGFP‐PNPT1 (green) in HeLa following the uptake. b) Association of internalized biotin‐miR‐21 with PNPT1 in HEK293T cells assessed by immunoprecipitation of cellular biotin‐miR‐21. c) Generation of a permanent PNPT1‐KO A549 cell line, and PNPT1 knockout in A549 cells was confirmed by Western blot analysis (upper) and immunofluorescence labeling (lower). d) Cy5‐miR‐21 failed to be delivered to mitochondria in the PNPT1‐KO A549 cells after 4 h incubation compared with WT A549, whereas the early uptake of Cy5‐miR‐21 by PNPT1‐KO A549 cells remained intact. e) Distribution of exogenous hcmv‐miR‐UL148D in mitochondria of WT and PNPT1‐KO A549 cells after incubation for various time points. The level of hcmv‐miR‐UL148D was quantified by RT‐qPCR. Scale bars: 10 µm. The experiments were repeated three times and samples in each time point of RT‐qPCR assay were triplicated. Statistical differences between samples were assessed by independent‐samples *t*‐test. Data with *P*‐value < 0.05 were considered statistically significant.

Recent studies have established PNPT1 as a transporter of various RNAs from the cytoplasm to mitochondria.^[^ ^39^, ^40^ ^]^ To test whether PNPT1 is involved in delivering the internalized miRNA via the miRNA phase separation pathway into mitochondria, we first generated HeLa cells that overexpressed Enhanced Green Fluorescent Protein (EGFP)‐PNPT1. HeLa cells were transfected with PNPT1‐EGFP‐expressing plasmid 48 h prior to the tracing experiment. To trace the internalized 5′‐Cy5‐miR‐21, EGFP‐PNPT1‐expressing cells were incubated with 50 nm 5′‐Cy5‐miR‐21 at 37 °C for 15 min in the presence of serum, and then washed with DMEM to remove unbound 5′‐Cy5‐miR‐21. Cells were then continuously incubated in new culture medium for various time points. As shown in **Figure** **5**a, more and more internalized 5′‐Cy5‐miR‐21 (red) was colocalized with PNPT1 (green) along the incubation course, implicating that PNPT1 may bind to the internalized 5′‐Cy5‐miR‐21 and deliver it to mitochondria. In line with this, immunoprecipitating the internalized miR‐21 tagged with biotin (biotin‐miR‐21) indicated the association of internalized biotin‐miR‐21 with PNPT1. In the immunoprecipitation experiment, cells were incubated with biotin‐miR‐21 for 12 h in the presence of serum, and then subjected to cell lysis and immunoprecipitation of biotin‐miR‐21. Western blot analysis showed that significantly more PNPT1 was pulled down by biotin‐miR‐21 compared to the control (Figure 5b).

To define the role of PNPT1 in mitochondria delivery of internalized miRNA, we generated a PNPT1‐deficient A549 cell line using the CRISPR‐Cas9 system. As shown in Figure 5c, Western blot (top) and immunofluorescence analysis (bottom) confirmed that PNPT1 was successfully knocked out in A549 cells. Uptake and intracellular localization of 5′‐Cy5‐miR‐21 clearly showed that the mitochondria delivery of internalized 5′‐Cy5‐miR‐21 was blocked in PNPT1‐KO A549 cells compared to that in WT A549 cells (Figure 5d), though the earlier internalization process of 5′‐Cy5‐miR‐21 in PNPT1‐KO A549 cells was not affected. Mitochondria isolation and qRT‐PCR assays further validated the role of PNPT1 in delivering the internalized miRNA to mitochondria. In this experiment, control A549 and PNPT1‐KO A549 cells were incubated with hcmv‐miR‐UL148D in DMEM containing 10% FBS. After incubation for 0, 1, 2, and 4 h, cells were washed off, harvested, and subjected to mitochondria isolation. After extraction of total RNAs, the level of hcmv‐miR‐UL148D in both mitochondria and mitochondria‐depleted cellular fractions was assessed by RT‐qPCR. As shown in Figure 5e, hcmv‐miR‐UL148D was equally internalized into control A549 and PNPT1‐KO A549 cells after short incubation (<1 h); however, the distribution of hcmv‐miR‐UL148D in two cell types was significantly different after 2 h incubation. In control A549 cells, more than 30% of internalized hcmv‐miR‐UL148D was detected in mitochondria after 2 h incubation. By contrast, less than 10% of internalized hcmv‐miR‐UL148D was detected in mitochondria from PNPT1‐knockout A549 cells after 2 h incubation. Taken together, these results suggest that PNPT1 deficiency disrupts the transport of internalized miRNAs into mitochondria though it has no impact on the uptake of miRNA nanoparticles by recipient cells.

### 2.4 The Internalized miR‐21 Upregulates Mitochondrial Cytochrome b in HEK293T Cells

**Figure 6:**
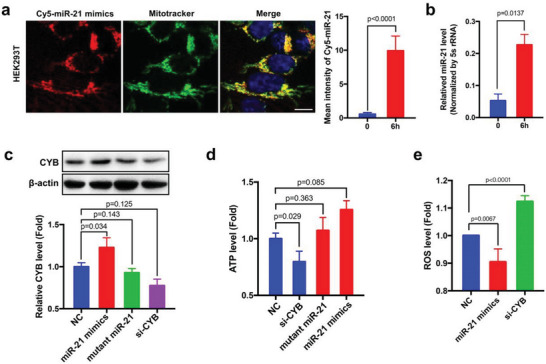
The internalized miR‐21 increases mitochondrial cytochrome b level in the recipient cells. a) Delivery of the internalized Cy5‐miR‐21 mimics to mitochondria in HEK293T cells after 6 h uptake mediated by the miRNA nanoparticle pathway. Scale bar: 10 µm. b) Delivery of the internalized miR‐21 mimics to HEK293T mitochondria after 6 h uptake via the miRNA nanoparticle pathway assessed by RT‐qPCR. c) Effect of internalized miR‐21 mimics and si‐CYB on mitochondrial CYB expression level. d) ATP production in HEK293T cells following the uptake of miR‐21 mimic, miR‐21 mutant, si‐CYB or control oligonucleotide (NC). e) Mitochondrial ROS level in HEK293T cells following uptake of miR‐21 mimic, si‐CYB or NC. In fluorescence image quantification analysis, 10–12 image fields from 3 independent experiments were analyzed. In RT‐qPCR and Western blot assay, the experiments were repeated three times. Data in the experiment were compared by independent‐samples *t*‐test. Data with *P*‐value < 0.05 were considered statistically significant.

As our results show that intact miRNAs can enter the cells via phase separation in the presence of serum and subsequently delivered into mitochondria by PNPT1, we next ask whether these miRNAs are functionally active. In this experiment, HEK293T cells were incubated with miR‐21 mimics in the presence of 10% FBS for 48 h. As previous study by Li et al. reported that miR‐21 could upregulate mitochondrial CYB in a nonclassic miRNA functional manner,^[^ ^41^ ^]^ we examined the level and function of mitochondria CYB following uptake of miR‐21 mimics. As expected, fluorescence imaging and RT‐qPCR assay of the internalized 5′‐Cy5‐miR‐21 mimics (**Figure** **6**a) and miR‐21 mimics (Figure 6b) showed that internalized miR‐21 was predominantly delivered to mitochondria of HEK293T cells. Served as a control, siRNA against CYB (si‐CYB, the sequence was listed in Extended data, Table S1, Supporting Information) was used to knock down CYB expression. HEK293T cells were incubated with si‐CYB in the same way to miR‐21 mimic for uptake of si‐CYB. Western blot analysis of HEK293T indicated that mitochondria delivery of miR‐21 mimics increased mitochondrial CYB level (Figure 6c), which is in agreement with a previous finding that miR‐21 enhances translation efficiency of CYB in mitochondria.^[^ ^41^ ^]^ Similarly, incubating HEK293T cells with CYB siRNA in the presence of 10% FBS resulted in a marked CYB reduction.

As an essential component of respiratory complex III in mitochondria, CYB is positively associated with the production of cellular ATP.^[^ ^42^ ^]^ Given that mitochondria CYB was upregulated or reduced by incubating cells with miR‐21 mimic or CYB siRNA in the presence of serum, respectively, we next examined whether the cellular ATP level was altered accordingly. As shown in Figure 6d, the cellular ATP level in HEK293T cells was increased by miR‐21 but decreased by CYB siRNA. As mitochondria CYB has been reported to be involved in cellular ROS reduction,^[^ ^42^ ^]^ we next measured the cellular ROS level following incubation with miR‐21 mimics or CYB siRNA. The results showed that CYB siRNA markedly increased cellular ROS levels whereas miR‐21 mimics decreased cellular ROS levels (Figure 6e). These results collectively suggest that internalized miR‐21 via serum‐mediated miRNA nanoparticle pathway is intact and functionally active in the mitochondria of recipient cells.

## 3 Discussion

In the present study, we demonstrate a novel RNA phase separation‐mediated pathway for miRNA uptake, and that following this unique uptake process, the internalized miRNAs are delivered predominantly into mitochondria via PNPT1.

Our results described a previously unrecognized uptake pathway for extracellular small RNAs particularly miRNAs by various cells, in which the formation of nanoparticles through RNA phase separation in the presence of serum cationic protein is the key. Previous studies showed that large RNA nanoparticles (≈500 nm) could be formed when RNAs were mixed with protein or peptide with strong positive charge.^[^ ^43^ ^]^ Although the mechanism remains unknown, Law et al. showed that RNA drug could enter the recipient cells after forming large nanoparticles by mixing with a positively charged R9, and the uptake of RNA drug in nanoparticles was enhanced after reducing the size of nanoparticle through sonication.^[^ ^43^ ^]^ Different from the size of small RNA nanoparticles we observed in this study, the large size of RNA particles they detected may be due to the larger of RNA molecule and higher concentration of strong positive charge peptide. Indeed, when the ratio of R9 peptide versus RNA decreased, the size of RNA microparticles reduced.^[^ ^43^ ^]^ Our results also suggest that the positive charge of serum cationic proteins but not protein structure controls the miRNA phase separation since depleting cationic proteins in serum prevents the formation of miRNA nanoparticles whereas serum protein denature fails to affect miRNA nanoparticle production.

Different from simply membrane fusion, this unique uptake mediated by small RNA phase separation is an active process, which requires ATP and strong membrane fluidity. Depleting intracellular ATP or decreasing temperature completely blocked miRNA uptake. In line with this, such RNA nanoparticle‐mediated miRNA uptake requires a dynamic arrangement of cellular microfilament network of recipient cells. We found that rottlerin strongly prevented the internalization of 5′‐Cy5‐miR‐29a (Figure 1b,d), suggesting that miRNA uptake via miRNA nanoparticles is through a mechanism similar to fluid phase macropinocytosis.^[^ ^25^, ^44^ ^]^ Given important role of extracellular miRNA in the recipient cells, extensive studies have been carried out to identify the pathway for extracellular miRNA uptake. These studies have demonstrated that extracellular miRNAs enter mammalian cells through multiple mechanisms, such as exosome‐mediated endocytosis, micropinocytosis or macropinocytosis, and the internalization process mediated by various RNA‐binding proteins^[^ ^16^, ^17^, ^18^, ^21^ ^]^ and lipoproteins.^[^ ^19^, ^20^, ^21^, ^45^ ^]^ As small RNA phase separation mediated by serum cationic protein is not dependent upon exosomes or specific RNA binding proteins, it may serve as a general uptake pathway for various small RNAs, especially those not encapsulated in exosomes or associated with RNA‐binding protein or lipoproteins.

Different from the delivery of miRNAs via Lipotransfection, in which the majority of delivered miRNAs were accumulated at lysosomes, the internalized miRNAs via small RNA nanoparticle pathway were mainly sorted to mitochondria. This finding suggests that mitochondria may serve as an important subcellular organelle for the storage of extracellular miRNAs, especially the miRNAs internalized into the recipient cells via the small RNA nanoparticle pathway. Through colocalization analysis and gene silence assay, we identified mitochondria‐associated PNPT1 as a transporter for delivering internalized miRNA into mitochondria. As an RNA‐binding protein associated with mitochondria, PNPT1 plays multiple roles in RNA transport and degradation.^[^ ^40^, ^46^ ^]^ Wang et al. reported that PNPT1 was involved in transport of various RNAs including miRNAs to mitochondria, in which it controls mitochondria RNA homeostasis.^[^ ^45^ ^]^ As a highly conserved polynucleotide phosphorylase, PNPT1 also plays an essential role in preventing the leakage of mitochondria double‐stranded RNAs into the cytoplasm.^[^ ^39^ ^]^ In line with this, our results show that PNPT1 is responsible for selectively delivering the internalized miRNAs via RNA nanoparticle‐mediated uptake pathway to mitochondria. Although PNPT1 also possesses activity in cleaving single‐stranded RNA,^[^ ^47^ ^]^ both RT‐qPCR and functional assay in the present study suggest that at least some extracellular miRNAs internalized through small RNA nanoparticle mechanism are intact, and can regulate the expression of target genes in mitochondria.

In summary, our studies reveal a general miRNA uptake pathway based on serum cationic protein‐mediated RNA phase separation, and a preferential transport of these internalized miRNA into mitochondria modulated by PNPT1. Given that small RNA nanoparticle pathway allows rapid uptake of miRNAs in bulk and transports the internalized miRNAs mainly into mammalian cell mitochondria not lysosomes, it may provide a novel mechanism for efficiently delivering large amount of functional miRNAs or RNA drugs in the future.

## 4 Experimental Section

### Reagents, Cell Lines, and Culture Conditions

The reagents used in this study were obtained from those sources: MitoTracker Green FM (Invitrogen; Carlsbad, CA), LysoTracker Green DND‐26 (Invitrogen), ER‐Tracker Green (Invitrogen), Hoechst 33342 (Invitrogen), cytochalasin B (APExBio, A8487), rottlerin (Abcam, Ab120377), dynasore (MedChemExpress, HY‐15304), nocodazole (APExBio, A8487), EIPA (MedChemExpress, HY‐101840), Dibucaine (MedChemExpress, HY‐B0552), Sphingosine (StredMarq, SIH‐202), RNase inhibitor Ribonucleoside Vanadyl Complexes (RVC) (Beyotime), and TRIzol (Sigma‐Aldrich). HeLa, A549, HEK293T, SGC‐7901, and U87MG cell lines were purchased from American Type Culture Collection (ATCC; Manassas, VA). MIN6 cell line was donated by Prof. Xiao Han at School of Basic Medical Science, Nanjing Medical University. SGC‐7901 cells were cultured in RPMI‐1640 (Gibco; Carlsbad, CA) supplemented with 10% (v/v) FBS (Genial; Brighton, CO). MIN6 cells were maintained in RPMI‐1640 contained with 1% l‐glutamine and 15 mm HEPES, supplemented with 15% (v/v) FBS and 0.1% 2‐hydroxy‐1‐ethanethiol (Sigma‐Aldrich). All other cells were cultured in DMEM (Gibco; Carlsbad, CA) supplemented with 10% (v/v) FBS at 37 °C under 5% CO_2_.

### Antibodies and Artificial Synthetic RNA Molecules

Antibodies against GAPDH and *β*‐actin, as well as goat anti‐rabbit and goat anti‐mouse IgG, were purchased from Cell Signaling Technology (Danvers, MA), antibody against mt‐CYB was obtained from Affinity Biosciences (Beijing, China). Antibody against PNPT1 was purchased from Santa Cruz Biotechnology (Santa Cruz, CA). Antibody against ATP5A1 was obtained from Proteintech (Wuhan, China). The second antibody donkey anti‐mouse IgG Alexa Fluor 488 and donkey anti‐rabbit Alexa Fluor 594 were purchased by Invitrogen. The miRNAs, miRNA mimics, siRNAs, single‐strand RNAs, double‐strand RNAs, and control RNAs were obtained via chemical synthesis (Biosyntech; Suzhou, China).

### Fluorescence Imaging and Quantitative Analysis

Three fluorescence systems were used in the experiment: two‐photon laser confocal microscope (Leica TCS SP8‐MP, WZ, German) for presentation of subcellular structure, High‐Content Screening System (PerkinElmer Operetta, MA, USA) for providing long period live cell imaging and an inverted laser confocal microscope (Zeiss LSM 880 with Airyscan, Oberkochen, Germany) for high quality confocal image. In living cell fluorescence imaging, all miRNAs (for cell incubation or transfection) were used at 50 nm. The concentrations and incubation time of MitoTracker Green FM, LysoTracker Green DND‐26, ER‐Tracker Green, Hoechst 33342, cytochalasin B, rottlerin, dynasore, nocodazole, EIPA, Dibucaine, and Sphingosine were listed in Extended data, Table S2 of the Supporting Information. Cell transfection with small RNAs was according to manufacturer's protocol. To observe cells on upright microscope, cells were prepared on glass‐bottom dish. All cells were living when images were taken. Fluorescence images taken by Operetta High‐Content Screening System were analyzed by Columbus 2.4.1, a cellular imaging and analysis software provided by PerkinElmer. Fluorescence intensity of images taken by Leica TCS SP8‐MP was analyzed by Adobe Photoshop. The fluorescence intensity data were shown in Figure 1a, 1b, 3e, 3f, 4d, and 6a.

### Quantification of miRNAs in Mitochondria and Whole Cell Lysate by RT‐qPCR

For quantification of miRNAs in cell lysate, cells were cultured on 12‐well plate up to 80% confluence. Synthetic miRNAs (50 nm) were added into wells under different conditions. In 4 °C group, culture medium was precooled at 4 °C. For cells to deplete ATP or treated with various drugs, cells were pretreated with oligomycin (ATP synthase inhibitor, Abcam, Cambridge, UK) or other drugs prior to adding miRNA. The incubation time and concentration of drugs were listed in Extended data, Table S2 of the Supporting Information. For heat treatment of serum, MV‐free FBS was preheated in 60 °C for 30 min. To prevent RNA degradation, all culture medium were added 2.5% (v/v) RNase inhibitor RVC to block RNase activity. After 3 washes with PBS, cell total RNA was extracted using TRIzol according to manufacturer's protocol. Reverse transcription by AMV (Takara Biomedical Technology, Beijing, China) was then performed and level of miRNAs was further analyzed by RT‐qPCR on Roche Light cycler 96 Detection System (Roche, Auckland, New Zealand) using Takara Taq (Takara Biomedical Technology), dNTP, and TaqMan TM Probe (Applied Biosystems). The snRNA probe U6 was used to normalize each sample. For quantification of miRNAs in mitochondria, cells were harvested using 0.25% trypsin (Gibco, Carlsbad, CA). Mitochondria were isolated using Mitochondria Isolation Kit (Beyotime). Briefly, 1 mL mitochondria separation buffer was added into cells. After gently pipetting to resuspend the cells, a Dounce Tissue Grinder was used to break the cells and release mitochondria. Cell mixture was transferred into a 1.5 mL centrifuge tube and centrifuged (1000 × *g*, 4 °C) for 15 min. The supernatant was transferred into a new 1.5 mL centrifuge tube and centrifuged (3500 × *g*, 4 °C) for another 15 min. The pellet was collected as mitochondria. The miRNA levels in mitochondria were quantified by RT‐qPCR using TaqMan probe. The 5S rRNA (sequence was listed in Extended data, Table S1, Supporting Information) and SYBR system were used to normalize each sample in mitochondria and cells.

### Nanoparticle Formation, Separation, and Identification

This experiment was performed on Nanosight NS300 (Malvern PANalytical, England) to test the diameter of nanoparticles. Prior to the test, serum was ultracentrifuged (120 000 × *g*, 4 °C, 2 h) and filtered (using a 100 kD cutoff ultrafiltration filter, 4000 × *g*, 20 min) to deplete MVs.^[ [^ ^8^ ^,35]^ 50 nm synthetic miR‐29a was then mixed with DMEM or DMEM containing 1%, 3% or 10% MV‐free FBS for 5 min at room temperature to form nanoparticles. To separate nanoparticle‐associated miR‐29a from free miR‐29a following miRNA phase separation in the presence of MV‐free FBS, the miRNA‐serum mixture was separated by centrifugation (4000 × *g*, 20 min) with a 100 kD cutoff filter. The miR‐29a in nanoparticles (NP‐miRNAs) was collected in the upper chamber while free miR‐29a was collected in the lower part. The miR‐29a in each fraction was extracted by TRIzol and quantified by RT‐qPCR. NP‐miR‐29a and free miR‐29a were adjusted to the same concentration prior to incubation with cells. To calculate miR‐29a level, an miR‐29a standard curve was established. As an exogenous miRNA, synthetic hcmv‐miR‐148D was also used in the experiment and its partition in nanoparticle and nanoparticle‐free fraction or uptake by recipient cells was quantified using the same method described above. For fluorescence assay, cells were precultured in serum‐free DMEM for 30 min. For time course assay, only NP‐Cy5‐miR‐29a and free‐Cy5‐miR‐29a from 10% MV‐free FBS group were used.

### Ion Exchange Chromatography

Econo‐Column (Bio‐Rad; Herculs, CA) and SP Sepharose Fast Flow (GE; Boston, MA) were used to deplete serum cationic proteins. Briefly, pipetted 5 mL of the SP Sepharose beads suspension into the column, opened the valve of the column to let the buffer drain out driven by gravity. Next, balanced the column with 1 m KCl for 5 min. 10% MV‐free FBS was gently loaded into the column. Cationic protein‐depleted serum was then collected in the outflow fraction.

### Immunofluorescence Assay

In immunofluorescence assay, cells were seeded on glass‐bottom dish. Mitochondria were stained with MitoTracker Red CMXRos (500 nm, 45 min). Samples were fixed by 4% paraformaldehyde (Beyotime) for 10 min at room temperature. To stain mitochondria markers, cells were treated with 0.05% Triton X‐100 in PBS containing 5% bovine serum albumin (BSA) (Sangon Biotech) for 30 min for cell permeabilization. After treatment with 5% BSA blocking solution for 30 min, cells were incubated with PNPT1 antibody and ATP5A1 (diluted 1:100 in blocking solution) for 3 h at room temperature. After extensive washing, cells were then incubated with donkey anti‐mouse IgG Alexa Fluor 488 (Invitrogen) and donkey anti‐rabbit IgG Alexa Fluor 594 (Invitrogen) (diluted 1:1000 in blocking solution) for 1 h at room temperature. Prior to embedding, DAPI (Beyotime) was added to stain cell nucleus.

### RNA Immunoprecipitation

HEK293T cells cultured on 6 cm dish with ≈80% confluence were incubated with or without biotin‐labeled miR‐21 for 12 h. After 3 washes with PBS, precooled RIP buffer I (10 mm Tris‐HCl, pH7.5, 1 mm EDTA, 1 m NaCl, 0.01% Tween‐20) was added into dishes. Cell lysate was collected 5 min later and then mixed with Streptavidin magnetic beads (MedChemExpress, NJ, USA) to pull down the complex of biotin‐miR‐21 and the associated proteins. The mixture was gently shaken at 4 °C for 2 h. After three washes with PBS, proteins associated with biotin‐miR‐21 were then eluted by Radio Immunoprecipitation Assay (RIPA) lysate (Beyotime). Protein level was examined by Western blot.

### PNPT1‐KO Cell Line Construction

PNPT1 gene was knock out in A549 cell using the CRISPR‐Cas9 System. The gRNA:ATAGTGCTCGCACTTGCAAC (designed in http://crispr.mit.edu) was linked into the CRISPR Cas9 Plasmid (Genscript; Nanjing, China) and transformed into competent cell (Trelief 5*α*, Tsingke; Beijing, China). Harvested plasmids after 16 h cultivation were transfected into the cells using lipofectamine 3000 (Invitrogen; Carlsbad, CA) according to manufacturer's protocol. Cells were then screened out by 50 µm puromycin (Sangon Biotech) and seeded onto 96‐well plate to cultivate monoclonal cell. The monoclonal cells were selected through subculture and PNPT1 protein expression was examined by Western blot.

### Western Blot Assay

Cells were harvested by RIPA lysis buffer and the cell debris were depleted after 12 000 × *g* centrifuge at 4 °C. The supernatant was boiled at 100 °C for 5 min before loaded into 10% (for PNPT1) or 12% (for cytochrome b) polyacrylamide gel. Proteins resolved by SDS‐PAGE were transferred onto the polyvinylidene fluoride membranes. The membranes were blocked for 1 h and incubated with primary antibodies against PNPT1, GAPDH, CYB or *β*‐actin antibody (1:1000) at 4 °C for 6 h followed by six washes and incubation with secondary antibodies goat anti‐mouse IgG‐HRP or goat anti‐rabbit IgG‐HRP. Protein band intensities were quantified using ImageJ 1.44p software. Uncropped scans of all gels are provided in Source data file with this paper.

### Cell ATP Detection and Mitochondrial Reactive Oxygen Species (mt‐ROS) Detection

To detect cellular ATP production, cells were cultured on 12‐well plate. After incubation with synthetic control oligonucleotide, miR‐21 mimics, miR‐21 mutant or CYB siRNA (si‐CYB) for 48 h, cellular ATP production level relative light unit was detected by luminometer using ATP Assay Kit according to the manufacture's protocol (Beyotime). To test the level of mitochondria ROS, HEK293T cells were seeded into 24‐well plate and incubated with synthetic control oligonucleotide, miR‐21 mimics, miR‐21 oligonucleotide orsi‐CYB for 48 h. After washing with HBSS (Beyotime), cells were incubated with 500 nm MitoSox Red (Invitrogen) in prewarmed HBSS for 30 min. Cells were harvested by 0.25% trypsin and the ROS level was measured by flow cytometry.

### Statistical Analysis

Data derived from three independent experiments were presented as mean ± SD (standard deviation). Statistical differences between samples or groups were assessed by two‐way ANOVA with Dunnett's multiple comparisons test (Figure 1a,c), paired *t*‐test (Figure 3e,f; Extended data, Figure S3, Supporting Information) or independent‐sample *t*‐test (others). All original data were supplied in the Supporting Information, with CT values, processing of standardizing and normalization of each independent replicates included. Statistical analyses and generation of graphics were performed using Microsoft Excel and GraphPad Prism 7. Data with *P*‐value < 0.05 were considered statistically significant.

## Conflict of Interest

The authors declare no conflict of interest.

## Author Contributions

J.L., W.L., J.L., and E.S. contributed equally to this work. K.Z., Y.Z., C.Z., and S.G. designed the experiments. J.L., W.L., J.L., A.S., H.L., W.R., X.J., N.X., W.W., and S.Q. performed the experiments and analyzed results. K.Z. and J.L. drafted the manuscript. All authors critically revised and approved the final version.

## Supporting information

Supporting Information

## Data Availability

The data that support the findings of this study are available from the corresponding author upon reasonable request.
