# Supplementary material for: A Novel Pathway of Functional microRNA Uptake and Mitochondria Delivery
Source: Adv Sci (Weinh). 2023 Jun 25;10(24):2300452. doi: 10.1002/advs.202300452 (PMC10460862; doi:10.1002/advs.202300452)
Supplement: Supplementary file 1 — Supporting Information [file ADVS-10-2300452-s001.pdf]

ADVANCED SCIENCE
Open Access

Supporting Information

for *Adv. Sci.*, DOI 10.1002/advs.202300452

A Novel Pathway of Functional microRNA Uptake and Mitochondria Delivery

Jiachen Liu, Weili Li, Jianfeng Li, Eli Song, Hongwei Liang, Weiwei Rong, Xinli Jiang, Nuo Xu, Wei Wang, Shuang Qu, Shouyong Gu, Yujing Zhang*, Chen- Yu Zhang* and Ke Zen**

Extended Data for

A novel pathway of functional microRNA uptake and mitochondria delivery

File list:

1. Extended Data | Table 1

2. Extended Data | Table 2

3. Extended Data | Figure S1

4. Extended Data | Figure S2

5. Extended Data | Figure S3

Extended Data | Table 1. Sequence of RNAs used in the experiment.

RNA	Sequence (5' to 3')
miR-29a	UAGCACCAUCUGAAAUCGGUUA
miR-122	UGGAGUGUGACAAUGGUGUUUG
hcmv-miR-UL148D	UCGUCCUCCCCUUCUUCACCG
5S rRNA qRT-PCR Primer	sense: GATCTCGTCTGATCTCGGAAGC<br>anti-sense: AAAGCCTACAGCACCCGGTATT
miR-1 mimics	sense: UGGAAUGUAAAGAAGUAUGUAU<br>anti-sense: UGGAAUGUAAAGAAGUAUGUAU
ds-miR-29a	sense: UAGCACCAUCUGAAAUCGGUUA<br>anti-sense: UAACCGAUUUCAGAUGGUGCUA
miR-21 mimics	sense: UAGCUUAUCAGACUGAUGUUGA<br>anti-sense: AACAUCAGUCUGAUAAGCUAUU
si-CYB	sense: CCACUAAGCCAAUCACUUUTT<br>anti-sense: AAAGUGAUUGGCUUAGUGGTT

Extended Data | Table 2. Concentration, treatment time and function of reagents used in the experiments.

Treatment	Working Concentration	Duration	Function
Oligomycin	10ng/mL	30 min	ATP depletion
Rottlerin	10μM	45 min	Fluid phase endocytosis inhibitor
Cytochalasin B	5uM	1 h	actin polymers formation inhibitor
Dynasore	30uM	30 min	GTPase inhibitor
Nocodazole	20nM	30 min	Microtubule inhibitor
EIPA	50μM	2 h	Sodium-hydrogen exchanger（NHE）inhibitor
Mito Tracker Green	1/2000	40 min	Mitochondria label
Mito Tracker Red CMXRos	500nM	45 min	Mitochondria label
Lyso Tracker Green	1/500	30 min	Lysosome label
ER Tracker Green	1/1000	30 min	ER label
Hoechst	1/1000	10 min	Cell nucleus label
Dibucain	0.05μM	2 h	Displace mitochondria surface charge

Sphingosine	20μM	2 h	Displace mitochondria surface charge
Mito Sox Red	500nM	30 min	Mitochondria ROS detection
Puromycin	10μg/mL	24 h	Cell selective agent
RVC RNase Inhibitor	5mM	10 min	Inhibit RNase activity

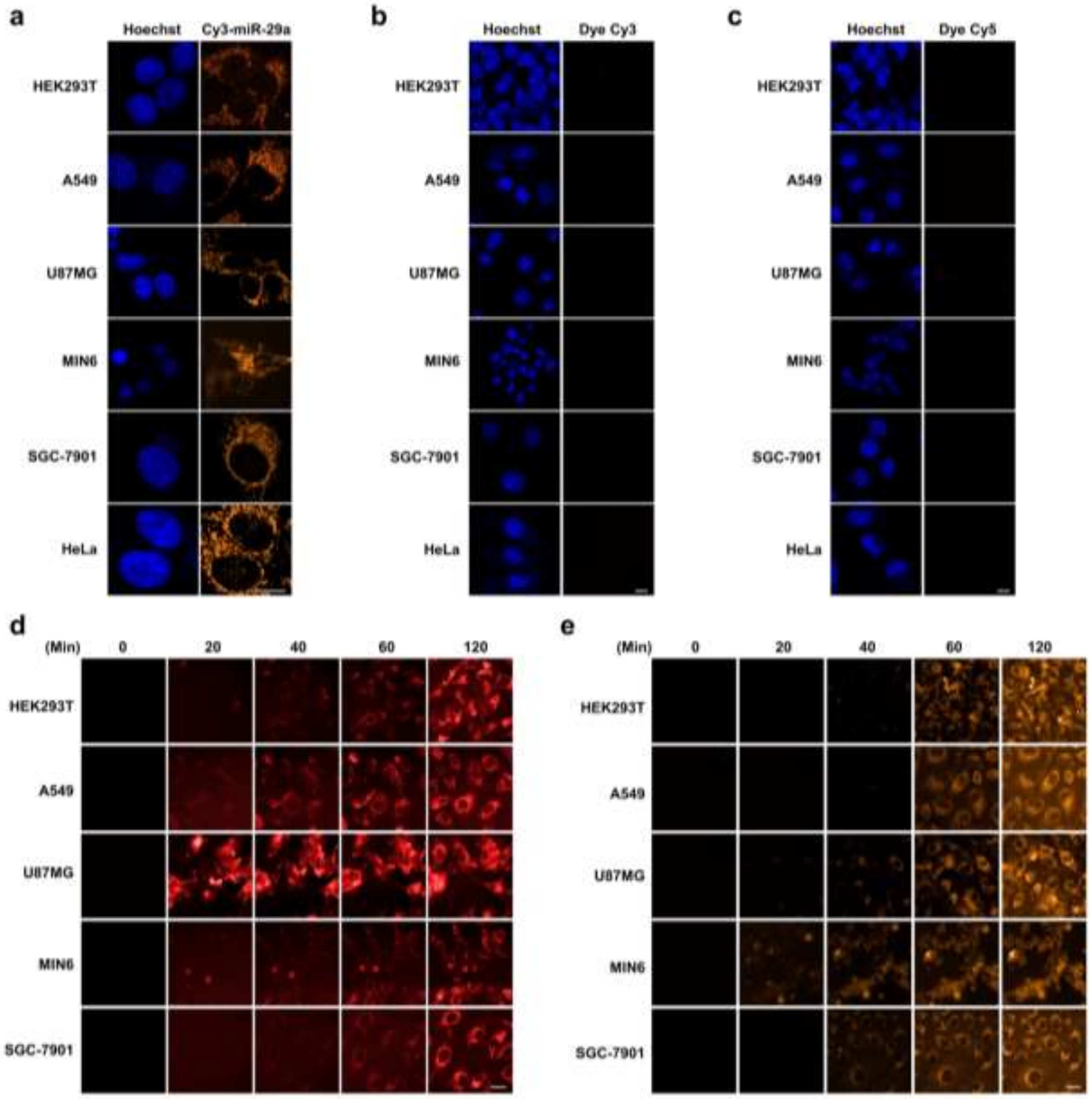

Extended Data | Figure 1. Uptake of miRNAs or fluorescent molecules by various cells. a, Uptake of 5’-Cy3-miR-29a in different cell lines. **b and c**, No uptake of Cy5 or Cy3 alone by different cell lines. **c and d**, The time-course of 5’-Cy5-miR-29a or 5’-Cy3-miR-29a uptakes by various cells. In panel a, b and c, scale bars, 10μm. In panel d, scale bar, 20μm.

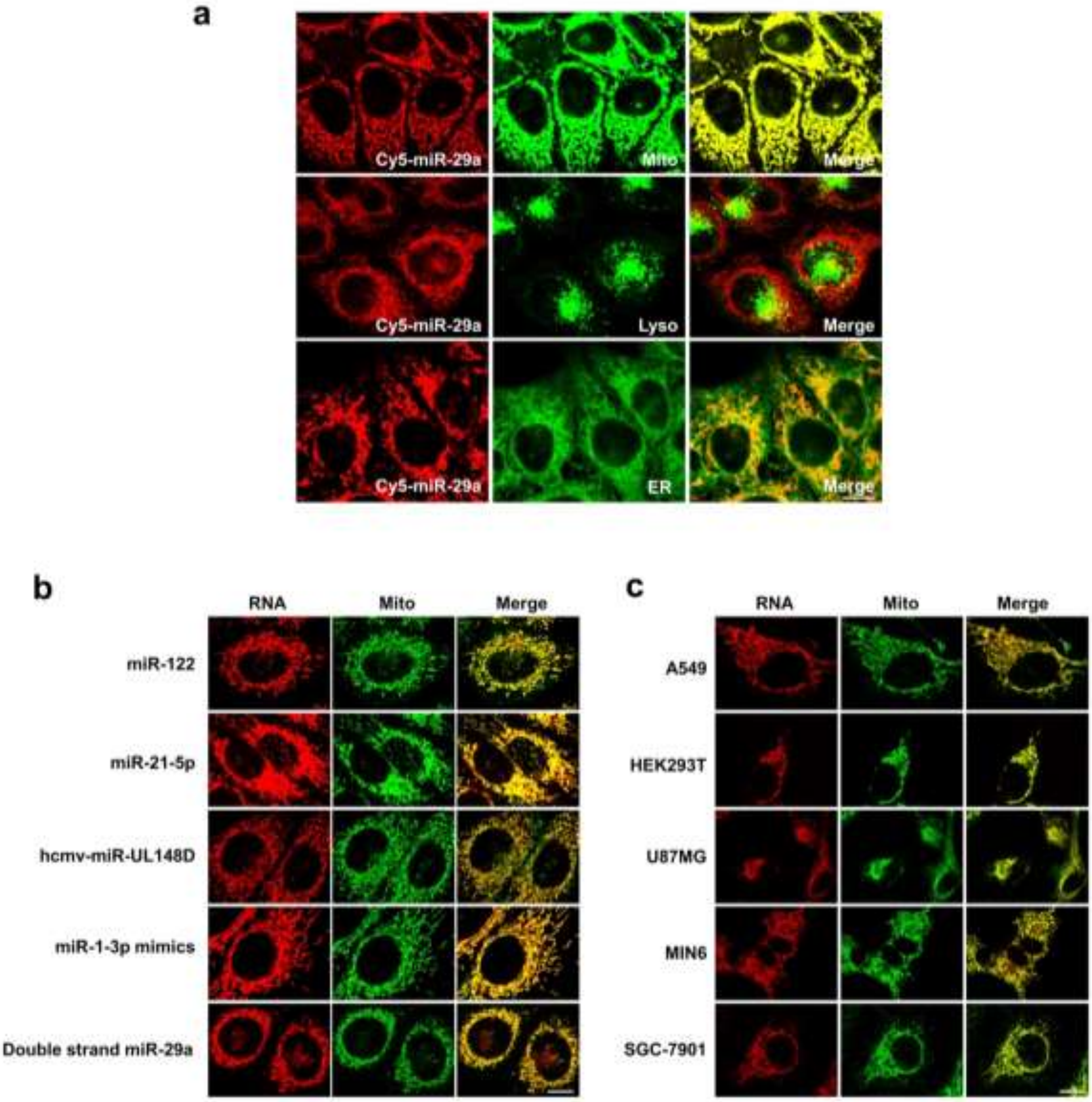

Extended Data | Figure 2. Preferential delivery of the internalized small RNAs via nanoparticle pathway into mitochondria in various cells. a, Distribution of internalized 5’-Cy5-miR-29a in mitochondria (mito), but not lysosomes (lyso) and endoplasmic reticulum (ER) in various cells after 6 h incubation. **b,** Colocalization of various internalized small RNAs with mitochondria marker in HeLa cells after 12 h incubation. **c**, Colocalization of 5’-Cy5-miR-29a with mitochondria marker in different cells after 12 h incubation. Scale bars, 10μm.

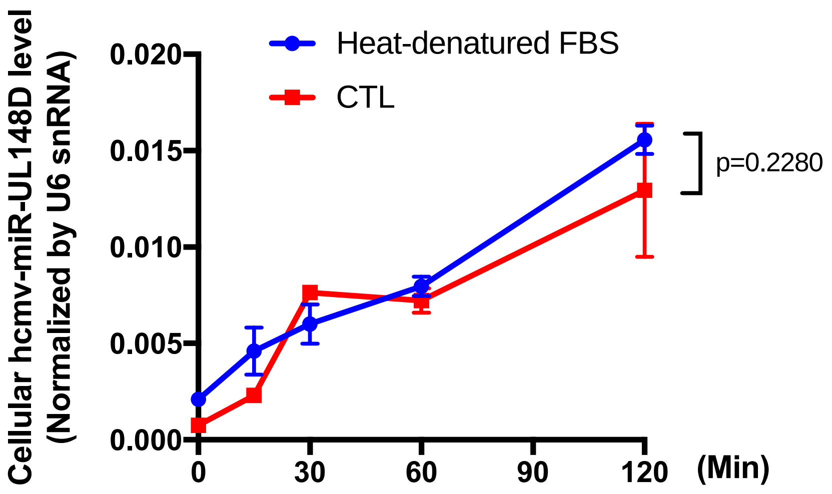

Extended Data | Figure 3. Uptake of hcmv-miR-UL148D by HeLa cells in the presence of 10% normal FBS (CTL) or heat-denatured FBS. The experiments were repeated 3 times and samples in each time point of RT-qPCR assay were triplicated. Statistical differences between groups was assessed by paired T-Test. The data were presented as mean ± SD.
